# A case of unintentional superior vena cava isolation and dormant conduction after right superior pulmonary vein pulsed field ablation

**DOI:** 10.1016/j.hrcr.2026.03.028

**Published:** 2026-04-09

**Authors:** Kazuya Murata, Yasuteru Yamauchi, Hirohumi Arai, Atsuhito Oda, Yuichiro Sagawa, Tetsuo Sasano

**Affiliations:** 1 Department of Cardiology, Japan Red Cross Yokohama City Bay Hospital, Yokohama City, Kanagawa, Japan; 2 Department of Cardiovascular Medicine, Institute of Science Tokyo, Bunkyo-ku, Tokyo, Japan

**Keywords:** Adenosine, Atrial fibrillation, Dormant conduction, Pulsed field ablation, Right superior pulmonary vein isolation, Superior vena cava, Unintended superior vena cava isolation

**Key Teaching Points**

• Pulsed field ablation delivered to the right superior pulmonary vein can unintentionally isolate the superior vena cava when the 2 structures are in close anatomical proximity.
• Even when the superior vena cava appears electrically isolated after incidental isolation, adenosine can unmask dormant conduction that may permit later reconnection.
• When non–pulmonary vein triggers from the superior vena cava are suspected or observed, adenosine testing can help confirm block completeness and guide targeted adjunctive ablation.

## Introduction

Previous studies have reported that radiofrequency (RF) ablation, cryoballoon ablation, or pulsed field ablation (PFA), targeting the right superior pulmonary vein (RSPV), may unintentionally lead to isolation of the superior vena cava (SVC).^1^, ^2^, ^3^ Several studies have shown that adenosine administration can unmask dormant conduction between the pulmonary veins and the left atrium as well as between the SVC and the right atrium after apparent electrical isolation, revealing viable but nonconducting myocardial fibers capable of reconnection.^4^, ^5^, ^6^, ^7^ However, to our knowledge, no reports have described adenosine-induced unmasking of dormant conduction in the SVC during unintended SVC isolation caused by PFA. This report describes adenosine-unmasked dormant conduction in the SVC after unintended SVC isolation during RSPV PFA.

## Case report

A 59-year-old man was referred for catheter ablation owing to recurrent palpitations associated with paroxysmal atrial fibrillation. 6 years earlier, he had undergone catheter ablation for atrial fibrillation at another hospital.

**Figure 1:**
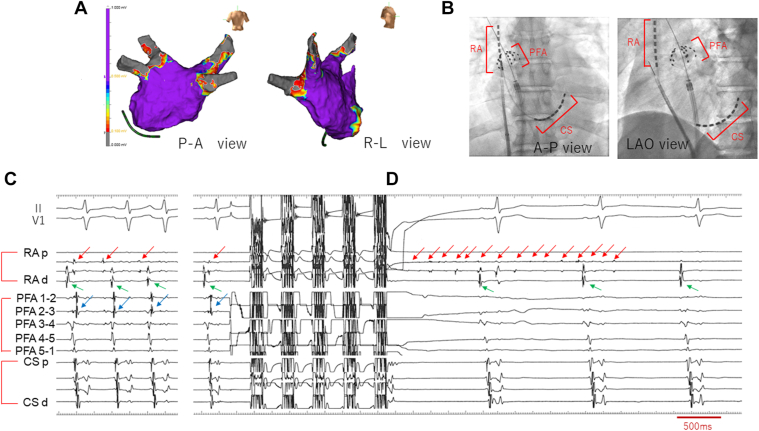
Unintentional isolation of the SVC. **A:** Pre-PFA left atrial voltage maps during right atrial pacing. Voltage is color-coded from *gray* (scar/low) to *purple* (normal). **B:** Catheter positions in the A-P and LAO views. A 20-lead electrode catheter is advanced from the right internal jugular vein into the CS, recording signals from the SVC, RA, and CS and displaying potentials from 16 electrodes. A PFA catheter is positioned in the RSPV to assess PV potentials. An intracardiac echocardiography probe is placed in the RA. **C:** Non-PV trigger from the SVC. The *red arrow* marks the SVC potential; the *green arrow* marks the RA potential; and the *blue arrow* marks the RSPV potential, indicating AF originating from the SVC. **D:** PFA delivery to the RSPV. After ablation, RSPV potentials are eliminated. The SVC is electrically isolated; AF activity persists transiently only within the SVC (*red arrow*), whereas atrial activity elsewhere is quiescent. AF = atrial fibrillation; A-P = anteroposterior; CS = coronary sinus; d = distal; LA = left atrium; LAO = left anterior oblique; p = proximal; P-A = posteroanterior; PFA = pulsed field ablation; PV = pulmonary vein; RA = right atrium; R-L = right-to-left; RSPV = right superior pulmonary vein; SVC = superior vena cava.

A second ablation procedure was performed, and high-density mapping of the left atrium using a multipolar catheter [Advisor HD Grid mapping catheter (Abbott, IL)] revealed reconnection of the RSPV (Figure 1A). Provocation with isoproterenol induced atrial fibrillation originating from the non–pulmonary vein (PV) foci, with ectopic firing identified in the SVC (Figure 1B and 1C).

Initially, the PFA catheter [FARAPULSE PFA system (Boston Scientific, MA)] was used to isolate the PVs and posterior wall of the left atrium. During the first basket-configuration PFA application targeting the RSPV, the SVC was unintentionally isolated (Figure 1D). Immediately thereafter, fibrillatory activity was recorded exclusively within the SVC, consistent with dissociated SVC arrhythmia (Figure 1D). PFA targeting the RSPV was delivered in the standard manner: 2 applications were delivered in the basket configuration, followed by 2 additional applications after catheter rotation; the same sequence was then repeated in the flower configuration.

**Figure 2:**
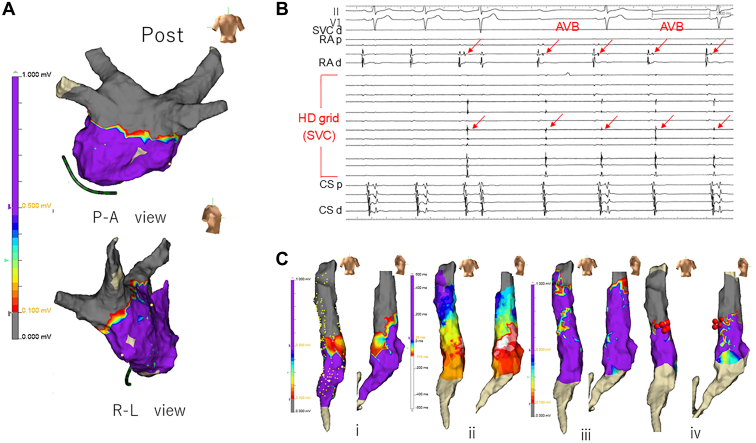
LA mapping, adenosine testing, and SVC isolation. **A:** Post-PFA left atrial voltage maps during right atrial pacing. Voltage is color-coded from *gray* (scar/low) to *purple* (normal). **B:** Adenosine test within the SVC using an HD Grid catheter. Transient atrioventricular block occurred after adenosine administration, during which SVC potentials (*red arrow*) have recurred. **C:** SVC maps in sinus rhythm. (i) Post-PFA SVC voltage map. (ii) Post-adenosine activation map. (iii) Post-adenosine voltage map. (iv) Post-RF SVC isolation voltage map. *Red tags* indicate RF lesions. Voltage is color-coded from *gray* to *purple*; activation time is color-coded from *white* (earliest) to *purple* (latest). AVB = atrioventricular block; CS = coronary sinus; d = distal; HD Grid catheter = Advisor HD Grid mapping catheter; LA = left atrium; p = proximal; P-A = posteroanterior; PFA = pulsed field ablation; RA = right atrium; R-L = right-to-left; RF = radiofrequency; SVC = superior vena cava.

Subsequent high-density mapping of the left atrium and SVC after PV and posterior wall isolation confirmed that in addition to the bilateral PVs and left atrial posterior wall (Figure 2A), the SVC remained electrically isolated. Consequently, isoproterenol infusion failed to induce non-PV triggers and no SVC reconnection was observed.

As the ectopic activity had previously originated in the SVC, 40 mg of adenosine was rapidly administered intravenously to evaluate dormant conduction (Figure 2B). Transient reconnection was observed, and conduction within the SVC resumed.

**Figure 3:**
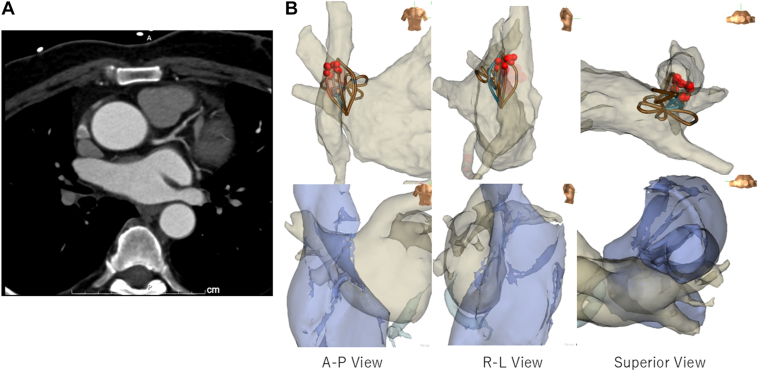
Anatomical relationship between the SVC and RSPV and ablation sites. **A:** Contrast-enhanced CT demonstrates the close proximity of the SVC and RSPV. **B:** Upper panels: 3-dimensional electroanatomic map shows PFA applications delivered in basket and flower configurations at the RSPV. *Red tags* denote RF applications. *Red*-shaded regions represent projection mapping of points located within 5 mm of the third electrode on the PFA catheter. Lower panels: 3-dimensional CT reconstruction; the *blue chamber* is the RA, and the *white-gray chamber* is the LA. A-P = anteroposterior; CT = computed tomography; LA = left atrium; PFA = pulsed-field ablation; RA = right atrium; RF = radiofrequency; R-L = right-to-left; RSPV = right superior pulmonary vein; SVC = superior vena cava.

Repeated high-density mapping of the SVC demonstrated a conduction block in all regions except the posterior wall (Figure 2C). Therefore, irrigated-RF ablation [TactiFlex ablation catheter (Abbott, IL)] was applied to the posterior wall of the SVC to achieve complete SVC isolation. A total of 8 RF applications were delivered point by point to the posterior SVC wall using a contact force of 5–10 g at 50 W for 5–8 seconds, with complete SVC isolation achieved after the second application (Figures 2C and 3B). No dormant conduction was observed after a second adenosine challenge. At 10-month follow-up, the patient remained free of atrial fibrillation.

## Discussion

SVC isolation has been recognized as a relevant adjunctive strategy in selected patients with atrial fibrillation, particularly in the presence of non-PV foci originating from the SVC.^8^^,^^9^ Although intentional SVC isolation has traditionally been performed with RF energy, recent reports indicate that unintentional SVC isolation can occur during RSPV isolation using RF, cryoballoon, or PFA.^1^, ^2^, ^3^ This phenomenon likely reflects the close anatomical relationship between the RSPV and the SVC wall.^9^^,^^10^ Tanaka et al^11^ reported that a spontaneous conduction block line between the right atrium and the SVC was observed in 49.5% of cases. When the RSPV and SVC are in close proximity and a spontaneous block exists outside their direct contact zone, SVC isolation may occur during RSPV ablation.

Yagishita et al^1^ and Ichihara et al^9^ demonstrated that energy delivered to the RSPV—either using RF or cryoballoon—can affect the neighboring SVC myocardial sleeves, leading to inadvertent isolation or conduction delay.

Similarly, Ogawa et al^3^ recently reported that PFA delivered to the RSPV can produce delayed conduction or even electrical isolation of the SVC without direct SVC application. In their series, the FARAPULSE catheter influenced the SVC, with a sensitivity of 72.7% and a specificity of 100% when the SVC-RSPV distance was ≤4.3 mm.^10^ In the present case, this distance was 2.6 mm (Figure 3).

Although the exact mechanism cannot be definitively established in these cases, the observed SVC block appears more consistent with a direct electric field–mediated effect of PFA than with collateral thermal injury. Recent data have shown that SVC involvement after RSPV PFA is strongly associated with RSPV-SVC proximity, supporting a distance-dependent field effect. In addition, the feasibility and high acute efficacy of intentional SVC isolation using a pentaspline PFA catheter further support the susceptibility of SVC myocardial sleeves to electroporation under clinically applied PFA conditions.^12^ In our case, the marked anatomical proximity between the RSPV and the SVC provides a plausible explanation for inadvertent electroporation of the adjacent SVC sleeve.

In this case, a definitive conclusion cannot be drawn because the conduction pattern before intentional SVC isolation could not be confirmed. However, after adenosine administration, reconnection was observed only in the posterior wall of the SVC. Because the anterior wall is relatively distant from the RSPV, the effect of the pulsed field delivered to the RSPV was likely minimal. These findings suggest that conduction block in the anterior wall may have already been present from the outset, with complete SVC isolation subsequently achieved by ablation of the posterior wall (Figure 2C).

The present case extends prior observations by showing that unintentional SVC isolation due to PFA may leave behind dormant conduction pathways, which can be revealed by adenosine administration. This aligns with the findings of Watanabe et al,^2^ who reported adenosine-induced SVC reconnection after cryoballoon ablation, implying the presence of viable yet functionally suppressed myocardial fibers. Although adenosine testing has been widely used to identify dormant conduction in PVs and SVCs, its use, particularly in unintentionally isolated SVCs, is not yet well established.

To our knowledge, this is the first report demonstrating that adenosine can unmask dormant conduction after unintentional SVC isolation during RSPV PFA, thereby guiding subsequent RF ablation to achieve durable SVC disconnection. When SVC isolation is not intentionally planned, isolation may be incomplete, and adenosine testing can help with the assessment of the SVC block completeness. Moreover, these findings underscore the need to consider SVC proximity and potential electrical responses when delivering RSPV-directed PFA.

In clinical practice, if the SVC is suspected to harbor non-PV triggers, SVC isolation should be considered. Alternatively, adenosine testing may be performed to verify the durability of SVC isolation after unintentional isolation. Because this is a single-case report, the clinical implications of routine adenosine testing after inadvertent SVC isolation require confirmation in larger studies.

## Conclusion

Although SVC isolation occurred unintentionally during PFA, adenosine testing unmasked dormant conduction, enabling the completion of durable SVC isolation with adjunctive RF ablation. This case underscores the value of adenosine testing when inadvertent SVC isolation is observed after RSPV ablation. Future studies should define the incidence of unintentional SVC isolation, evaluate the utility of routine adenosine testing, and establish standardized management strategies to improve durability and safety.

## Declaration of generative AI and AI-assisted technologies in the writing process

During the preparation of this work, the authors used ChatGPT (OpenAI) in order to refine English grammar and style. After using this tool/service, the authors reviewed and edited the content as needed and take full responsibility for the content of the publication.

## Disclosures

The authors have no conflicts of interest to disclose.

## References

[1] Yagishita A., Yamauchi Y., Hirao K. (2014). Superior vena cava isolation by right pulmonary vein ablation. Europace.

[2] Watanabe T., Hachiya H., Igarashi M., Kusa S., Iesaka Y. (2019). A case of bidirectional conduction block within the superior vena cava induced by cryoballoon pulmonary vein isolation. Pacing Clin Electrophysiol.

[3] Ogawa T., Kamakura T., Miyazaki Y., Kusano K. (2025). Superior vena cava isolation after pulsed field ablation of the right superior pulmonary vein. Heart Rhythm.

[4] Macle L., Khairy P., Weerasooriya R. (2015). Adenosine-guided pulmonary vein isolation for the treatment of paroxysmal atrial fibrillation: an international, multicentre, randomised superiority trial. Lancet.

[5] Yamada T., Murakami Y., Plumb V.J., Kay G.N. (2006). Adenosine can also improve the conduction between the superior vena cava and right atrium after isolation. J Cardiovasc Electrophysiol.

[6] Viles-Gonzalez J.F., Miller M.A., d’Avila A. (2012). Dormant conduction revealed by adenosine to guide electrical isolation of the superior vena cava. Europace.

[7] Miyazaki S., Taniguchi H., Komatsu Y. (2013). Clinical impact of adenosine triphosphate injection on arrhythmogenic superior vena cava in the context of atrial fibrillation ablation. Circ Arrhythm Electrophysiol.

[8] Miyazaki S., Takigawa M., Kusa S. (2014). Role of arrhythmogenic superior vena cava on atrial fibrillation. J Cardiovasc Electrophysiol.

[9] Ichihara N., Miyazaki S., Kuroi A. (2015). Impact of pulmonary vein isolation on superior vena cava potentials with a second-generation cryoballoon. J Cardiovasc Electrophysiol.

[10] Matsuura H., Kamakura T., Oshima T. (2025). Incidence and characteristics of superior vena cava impact after pulsed-field ablation of the right pulmonary veins. J Cardiovasc Electrophysiol.

[11] Tanaka Y., Takahashi A., Takagi T. (2018). Novel ablation strategy for isolating the superior vena cava using ultra high-resolution mapping. Circ J.

[12] Ollitrault P., Chaumont C., Font J. (2024). Superior vena cava isolation using a pentaspline pulsed-field ablation catheter: feasibility and safety in patients undergoing atrial fibrillation catheter ablation. Europace.

